# New Drugs, Appliances, and Things Medical

**Published:** 1890-08-23

**Authors:** 


### NEW DRUGS, APPLIANCES, AND THINGS MEDICAL.

[All preparations, appliances, novelties, etc., of which a notice is desired, should be sent for The Editor, to care of The Manager, 140, Strand, London, W.C.]

### BOVRIL.

The manufacturers of the above have forwarded to us samples for examination and report. We have subjected these to the usual tests and find them to answer fully to all that is claimed for them. In the prospectus accompanying the Bovril preparation, it is stated that Bovril is composed of (1st) beef dried and powdered; (2nd) this added to best extract of meat. We have here certainly one of the most powerful combinations of nitrogenous nutriment possible, for it is not only a stimulant (the meat extract), but also a highly nutritious food (the dried and powdered beef). Meat extract alone, as we have repeatedly stated, is very good as a fillip and restorative, but it does not contain any tissue-repairing material. Now Bovril does, and so fulfils the requirements of a concentrated meat food. In addition, we find that the flavour is decidedly pleasant, without that suspicion of singed roast beef that too many beef extracts and foods possess. Of the Bovril wine, lozenges, and the beef flour for soups we can also speak in terms of high praise. They are pleasant to the eye and taste, and are sure to be much appreciated. We are glad to notice that these excellent preparations are on sale at most hotels and public-houses. It is a good thing for a man, when he is tired and thirsty, to get a cup of pleasant and refreshing soup, the stimulating effects of which last much longer than those of alcohol, and do not leave any after-effects of an ill kind. The company deserve success for this cause alone if they have in any way lessened the tendency to drunkenness by supplying a drink which is at once refreshing, stimulating, and not mawkish or insipid. In conclusion, we may mention that the preparations can be had peptonized to suit patients whose digestive powers are still feeble from disease.

### FLORADOR.

In our notice last week it should have been stated that the award received by the Florador Food Company at the Cookery Competition Exhibition at Westminster Town Hall recently, was the only gold medal given for foods of all classes.

